# High-fat diets enhance and delay ursodeoxycholic acid absorption but elevate circulating hydrophobic bile salts

**DOI:** 10.3389/fphar.2023.1168144

**Published:** 2023-04-17

**Authors:** Liang Huang, Wei Wei, Xiaomei Huang, Xuejing Li, Haisha Liu, Lanlan Gui, Jinping Jiang, Linfei Wan, Xiangxiang Zhou, Jingsong Ding, Xuehua Jiang, Bikui Zhang, Ke Lan

**Affiliations:** ^1^ Key Laboratory of Drug-Targeting and Drug Delivery System of the Education Ministry and Sichuan Province, Sichuan Engineering Laboratory for Plant-Sourced Drug and Sichuan Research Center for Drug Precision Industrial Technology, West China School of Pharmacy, Sichuan University, Chengdu, China; ^2^ West China Second University Hospital, Sichuan University, Chengdu, China; ^3^ Department of Phase1 Clinical Trial Research Center, Xiangya Boai Rehabilitation Hospital, Changsha, China; ^4^ Chengdu Cynogen Bio-pharmaceutical Tech Co, Ltd., Chengdu, China; ^5^ Xiangya School of Pharmaceutical Sciences, Central South University, Changsha, China; ^6^ Department of Pharmacy, The Second Xiangya Hospital, Central South University, Changsha, China

**Keywords:** ursodeoxycholic acid, food effect, pharmacokinetics, glycoursodeoxycholic acid, bile salts, bile acids

## Abstract

**Background:** Ursodeoxycholic acid (UDCA) is a natural drug essential for the treatment of cholestatic liver diseases. The food effects on the absorption of UDCA and the disposition of circulating bile salts remain unclear despite its widespread global uses. This study aims to investigate the effects of high-fat (HF) diets on the pharmacokinetics of UDCA and disclose how the circulated bile salts were simultaneously perturbed.

**Methods:** After an overnight fast, a cohort of 36 healthy subjects received a single oral dose (500 mg) of UDCA capsules, and another cohort of 31 healthy subjects received the same dose after consuming a 900 kcal HF meal. Blood samples were collected from 48 h pre-dose up to 72 h post-dose for pharmacokinetic assessment and bile acid profiling analysis.

**Results:** The HF diets significantly delayed the absorption of UDCA, with the T_max_ of UDCA and its major metabolite, glycoursodeoxycholic acid (GUDCA), changing from 3.3 h and 8.0 h in the fasting study to 4.5 h and 10.0 h in the fed study, respectively. The HF diets did not alter the C_max_ of UDCA and GUDCA but immediately led to a sharp increase in the plasma levels of endogenous bile salts including those hydrophobic ones. The AUC_0–72h_ of UDCA significantly increased from 25.4 μg h/mL in the fasting study to 30.8 μg h/mL in the fed study, while the AUC_0–72h_ of GUDCA showed no difference in both studies. As a result, the C_max_ of total UDCA (the sum of UDCA, GUDCA, and TUDCA) showed a significant elevation, while the AUC_0–72h_ of total UDCA showed a slight increase without significance in the fed study compared to the fasting study.

**Conclusion:** The HF diets delay UDCA absorption due to the extension of gastric empty time. Although UDCA absorption was slightly enhanced by the HF diets, the beneficial effect may be limited in consideration of the simultaneous elevation of circulating hydrophobic bile salts.

## 1 Introduction

Ursodeoxycholic acid (UDCA) is an important drug for the treatment of cholestatic liver diseases (Lazaridis et al., 2001). It occurs in small amounts in human bile and is enriched in bear bile, a Traditional Chinese Medicine with a history of more than 2000 years. UDCA was first marketed in Japan based on traditional experience as a choleretic drug in 1957. It was later developed for the treatments of cholesterol gallstones and cholestatic diseases in Japan, Europe, and the United States based on evidence from randomized clinical trials (Makino and Tanaka, 1998). Long-term treatment of UDCA may replace the cytotoxic hydrophobic bile salts and increase the biliary percentage of UDCA (Makino and Nakagawa, 1978; Podda et al., 1982; Stiehl et al., 1990; Beuers et al., 1992). Biliary enrichment of UDCA plays multiple pharmacological roles in solubilizing cholesterol, stimulating bile secretion, protecting biliary epithelial cells, inhibiting the apoptosis of hepatocytes, and regulating intrahepatic immunity (Paumgartner and Beuers, 2002). At present, the globally approved indications of UDCA are cholesterol gallstones and cholestatic liver diseases. It is the first-line treatment of primary biliary cirrhosis, also termed primary biliary cholangitis (Beuers et al., 2015a, b), a cholestatic disease caused by chronic autoimmune injury of small intrahepatic bile ducts.

**FIGURE 1:**
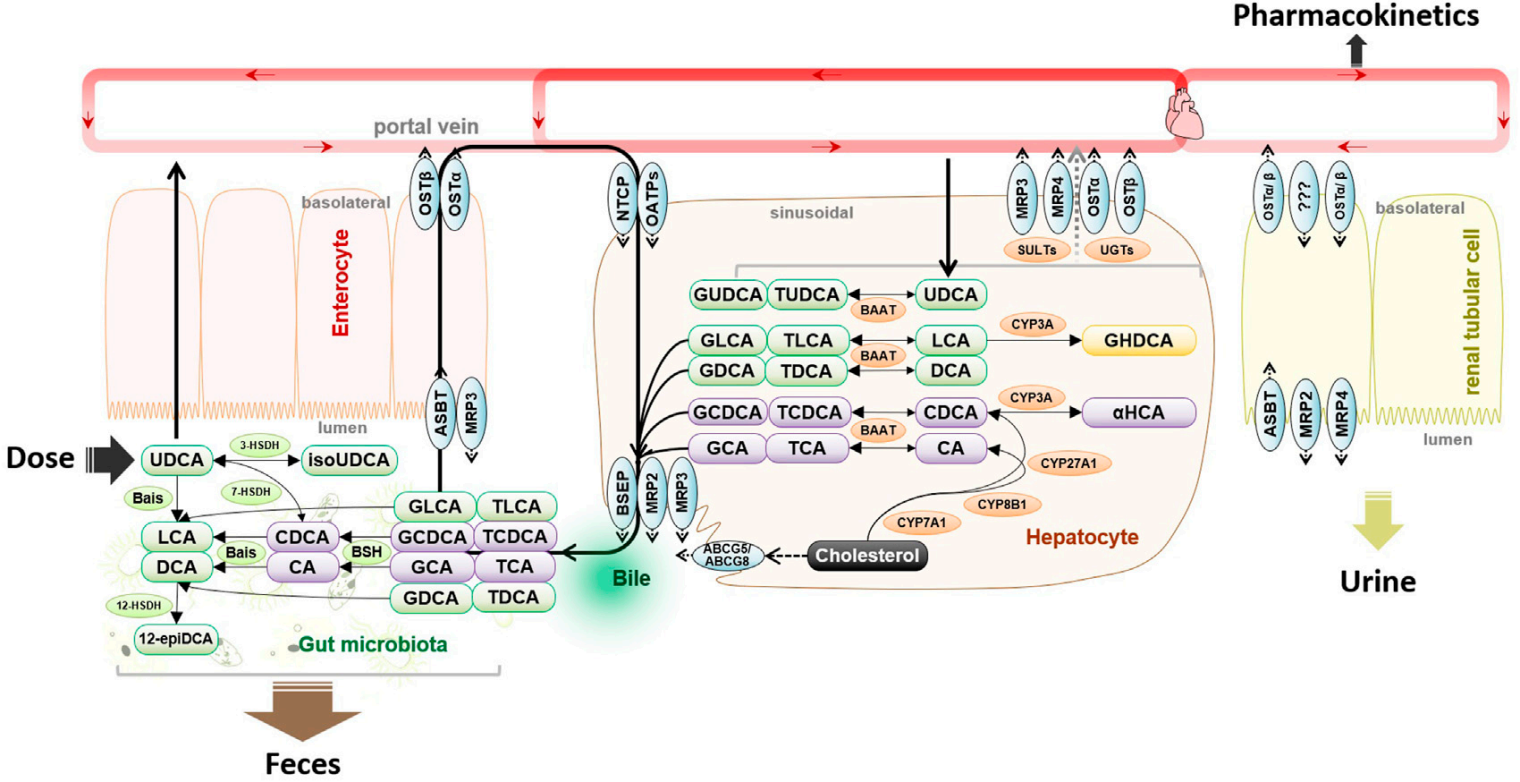
Metabolism and disposition of exogenous oral UDCA are intercrossed into the metabolic pathways of endogenous bile salts. Abbreviations: 3-HSDH, 3-hydroxysteroid dehydrogenase; 7-HSDH, 7-hydroxysteroid dehydrogenase; 12-epiDCA, 3α, 12β-dihydroxy-5β-cholan-24-oic acid; ASBT, apical sodium-dependent bile acid transporter; Bais, bile acid-inducible genes; BSEP, bile salt export pump; BSH, bile salt hydrolase; CA, cholic acid; CDCA, chenodeoxycholic acid; CYP3A, cytochrome P450 3A; DCA, deoxycholic acid; GCA, glycocholic acid; GCDCA, glycochenodeoxycholic acid; GDCA, glycodeoxycholic acid; GLCA, glycolithocholic acid; GUDCA, glycoursodeoxycholic acid; HDCA, hyodeoxycholic acid; isoUDCA, isoursodeoxycholic acid; LCA, lithocholic acid; NTCP, sodium taurocholate co-transporting polypeptide; OATP, organic anion transporting polypeptide; TCDCA, taurochenodeoxycholic acid; TDCA, taurodeoxycholic acid; TUDCA, tauroursodeoxycholic acid; and UDCA, ursodeoxycholic acid.

The clinical pharmacokinetics of UDCA is unique because its biological fate is dominated by the disposition mechanism of endogenous bile salts (Crosignani et al., 1996). As shown in Figure 1, the endogenous UDCA is epimerized from chenodeoxycholic acid (CDCA) in the lower gut by bacterial 7-hydroxysteroid dehydrogenase (7-HSDH), whose individual difference causes great variation of biliary UDCA percentage in populations. The amphipathic nature of UDCA demonstrated a pH-dependent solubility with micelle formation, leading to a sharp increase in solubility when micelle is formed at pH > 8.0 (Igimi and Carey, 1980). For this reason, the absorption of UDCA is usually incomplete because the gastrointestinal fluid is not alkaline enough to dissolve the clinical dose (Parquet et al., 1985; Stiehl et al., 1988). Upon uptake into hepatocytes, UDCA is conjugated extensively with glycine into glycoursodeoxycholic acid (GUDCA) and minorly with taurine into tauroursodeoxycholic acid (TUDCA) *via* peroxisomal bile acid CoA: amino acid N-acyltransferase (Batta et al., 1982). GUDCA and TUDCA are trapped into the bile acid pool *via* enterohepatic circulation driven by bile salt transporters. The enterohepatic circulation typically occurs about 2–3 times after each meal and about 4–12 cycles per day (Russell, 2003). As a result, UDCA has a high hepatic extraction approaching 70% and the plasma levels are reflective of the overflow of enterohepatic circulation into systemic circulation.

Gut flora participates in the disposition of UDCA as bile salts undergo extensive host-gut microbial co-metabolism (Ridlon et al., 2016). In the lower gut where gut flora colonizes, GUDCA and TUDCA that escape intestinal uptake are hydrolyzed by bile salt hydrolase (BSH). Along with the newly dissolved part of daily dose, UDCA may be epimerized into CDCA by 7-HSDH or into isoursodeoxycholic acid (isoUDCA) by bacterial 3-HSDH. In the colon, UDCA is converted by the bile acid-inducible gene-encoded enzymes into a water-insoluble metabolite, lithocholic acid (LCA). It is commonly accepted that fecal excretion of the undissolved UDCA and LCA plays a major role in UDCA elimination. A small part of LCA may be recovered and then 6α-hydroxylated into hyodeoxycholic acid (HDCA) by CYP3A (Araya and Wikvall, 1999). When the enterohepatic circulation is attenuated, renal excretion of the sulfate and glucuronide metabolites may play a complementary role in bile salt elimination (Raedsch et al., 1981; Gui et al., 2021). In summary, the disposition of UDCA is intercrossed into the host-gut microbial co-metabolism of bile salts, and the exogenous administration of UDCA has an incomplete oral absorption, a long retention time due to diet-related enterohepatic circulation, and an atypical elimination phase that is not suitable to be assessed by terminal half-life.

As an early-marketed drug, the clinical pharmacokinetics of UDCA is not fully understood with respect to the food effects. As a result, the label instructions of UDCA formulations are inconsistent between countries. In the United States, UDCA tablets (250 mg and 500 mg) are suggested to be taken with food, while UDCA capsules (250 mg) approved in Europe and China are recommended to be taken with some liquid without clear instruction of the food effects. On the other hand, it is generally accepted that UDCA may relieve symptoms of cholestasis such as pruritus *via* replacing the cytotoxic hydrophobic bile salts (Mela et al., 2003). It is also not fully understood how the circulated levels of hydrophobic bile salts are perturbed by UDCA given with or without food. For these reasons, we quantitatively analyzed the plasma levels of bile salts in the fasting and fed bioequivalence studies of UDCA capsules in healthy subjects to investigate the food effects on the absorption of UDCA and the disposition of circulating bile salts.

## 2 Materials and methods

### 2.1 Study design and subjects

Two randomized, single-dose, open-label, two-treatment period cross-over studies were performed in a single center to evaluate the fasting and fed bioequivalence of generic UDCA capsules (250 mg, Chengdu Cynogen Bio-pharmaceutical Tech. Co., Ltd., China) with reference to Ursofalk capsules (250 mg, Dr. Falk Pharma GmbH, Germany) in Chinese healthy volunteers. The study protocols were reviewed and approved by the local Medical Ethics Committee (Xiangya Boai Rehabilitation Hospital, Changsha, China), and the trials were registered on www.chinadrugtrials.org.cn (CTR20202360) according to the regulation of the Chinese National Medical Products Administration.

Eligible subjects were healthy men or women ≥ 18 years of age with a body mass index of 19 kg/m^2^–26 kg/m^2^. Female individuals who were not of childbearing potential were eligible for the study. Exclusion criteria included participation in any clinical trial within 3 months prior to the enrollment into this trial, clinically significant concomitant diseases (including acute or chronic gastrointestinal symptoms), medical history of acute cholecystitis, cholangitis, biliary obstruction, cholecystectomy, or clinically significant gastrointestinal surgery, clinically significant abnormal values in clinical laboratory screening tests, smoking (defined as > 5 cigarettes or the equivalent per day), drinking (defined as >14 units of alcohol per week), use of any prescription or non-prescription medication within 2 weeks prior to the first dosing, or ingestion of foods that affect drug metabolism within 1 week prior to the first dosing. In total, 36 and 32 subjects were enrolled in the fasting study and the fed study, respectively. One subject requested voluntary withdrawal from the fed study due to intolerance to the high-fat diet before drug administration at the first dosing visit.

### 2.2 Trial procedures and assessments

Both the fasting and the fed studies consisted of five visits: an informed consent visit, a screening visit, two single-dosing visits separated by a washout period of 28 days, and a follow-up visit. The fasting study and the fed study were sequentially performed with a one-week interval. During each dosing visit, subjects attended the clinical site 3 days prior to dosing and were asked not to smoke, perform strenuous physical exercise, take any medication, or consume alcohol, coffee, tea, chocolate, or other xanthine-containing beverages. During the in-hospital managements, a standard meal, which consisted of moderate amounts of rice, pork or beef, and vegetables, cooked in Chinese style, with slight variations of vegetables at each meal, was given to volunteers, and the baseline level of bile salts was measured at −48 h, −42 h, −36 h, −30 h, −24 h, −18 h, −12 h, −6 h, and 0 h pre-dose for both studies. For the fed study only, an HF meal (929.6 kcal, 28 energy percent [E%] carbohydrate, 54 E% fat, and 18 E% protein), which consisted of 1 hamburger, 2 chicken wings, 10 g unsalted butter, and 250 mL milk, was given to the subjects 30 min prior to the −48 h, −24 h, and 0 h baseline sample collection time points.

On the day before each dosing day, subjects of both studies received a standard dinner and subsequently fasted overnight. In the morning of the dosing day, subjects of the fasting study received a single oral dose of 500 mg ursodeoxycholic acid (two capsules) with 240 mL water, while subjects of the fed study received the high-fat breakfast 30 min prior to the same dosing procedure as the fasting study. For both studies, no other water consumption was allowed until 1 h after dosing and subjects were not allowed to lie supine for the first 2 h post-dose except for trial procedures. On each dosing day of both studies, blood samples for pharmacokinetic analysis were collected by venipuncture or cannulation at 0.25 h, 0.5 h, 1.0 h, 1.5 h, 2.0 h, 2.5 h, 3.0 h, 3.5 h, 4.0 h, 4.5 h, 5.0 h, 6.0 h, 7.0 h, 8.0 h, 10 h, 12 h, 16 h, 24 h, 36 h, 48 h, and 72 h post-dose. The standard lunch and dinner were, respectively, given at 4 h and 10 h post-dose. Blood samples were collected into the EDTA-K2 anticoagulated vacuum tubes. Plasma was separated by centrifuging at 2,000 g and 4°C for 10 min and stored at −80°C until analysis.

In this study, blood samples only from the subjects who received the reference formulation (Ursofalk capsules) in the fasting and fed bioequivalence studies were selected for quantitative analysis of the plasma levels of bile salts to exclude the effect of pharmaceutical factors.

### 2.3 Bioanalysis of UDCA, GUDCA, and TUDCA

Plasma concentrations of UDCA, GUDCA, and TUDCA were simultaneously measured by a fully validated LC-MS method on an ACQUITY ultra-performance liquid chromatograph coupled to a Xevo TQ-S mass spectrometer (Waters, Milford, MA, United States). In the 96-well plate, 50 µL plasma samples were spiked with 450 μL acetonitrile containing 100 ng/mL isotope-labeled standards (UDCA-D4 and GUDCA-D4), vortex-mixed at 1,500 rpm for 10 min, and centrifuged at 4°C at 4,600 g for 20 min. The supernatant (300 μL) was vacuum-evaporated at 50°C, reconstituted with 100 μL of water–acetonitrile–methanol mixture (50:40:10, v/v/v), and subjected to LC-MS analysis.

The mobile phases consisted of 1 mM ammonium acetate in water (mobile phase A) and a acetonitrile–methanol mixture (80:20, v/v) (mobile phase B). 5 μL of samples was injected onto a CORTECS C18 column (1.6 μm, 100 mm × 2.1 mm) (Waters, Milford, MA). The flow rate was 0.4 mL/min with the following gradient: 0.0–0.5 min (95% A), 0.5–0.7 min (95%–74% A), 0.7–2.0 min (74% A), 2.0–3.0 min (74%–68% A), 3.0–5.0 min (68%–62% A), 5.0–7.0 min (62%–52% A), 7.0–8.0 min (52%–45% A), 8.0–8.5 min (45%–5% A), 8.5–10.8 min (5% A), and 10.8–12.0 (95% A). The mass spectrometer was operated in the negative mode with a capillary voltage of 2.0 kV, source temperature of 150°C, desolvation temperature of 550°C, cone gas flow of 150 L/h, and desolvation gas flow of 900 L/h. The selected ion recording (SIR) of m/z 391 > 391 and m/z 395 > 395 with a collision energy (CE) of 36 eV was used to detect UDCA and UDCA-D4, multiple reaction monitoring (MRM) of m/z 448 > 74 and m/z 452 > 74 with a CE of 35 eV was used to detect GUDCA and GUDCA-D4, and MRM of m/z 498 > 80 with a CE of 60 eV was employed to detect TUDCA, respectively.

A set of eight calibration samples (50–10,000 ng/mL) prepared in phosphate buffers and three levels of quality control samples prepared in the pooled human plasma with known background levels of UDCA, GUDCA, and TUDCA were analyzed in each bioanalytical run. Data of a run were to be acceptable according to the criteria regulated in ICH M10. 7.4% and 7.8% samples of the fasting study and the fed study were repeatedly analyzed for incurred sample reanalysis (ISR). For the fasting study, 100%, 99.4%, and 100% ISR results were within ± 20% of the original results for UDCA, GUDCA, and TUDCA, respectively. For the fed study, 98.7% ISR results of UDCA, GUDCA, and TUDCA were within ± 20% of the original results.

### 2.4 Profiling of endogenous bile salts

Quantitative profiling of 26 endogenous bile salts is shown in Supplementary Table S1 and performed for the plasma samples collected at the dosing visit of Ursofalk capsules as described in our previous reports (Zhu et al., 2018; Zhang et al., 2019; Gui et al., 2021; Zeng et al., 2021). In brief, 5 μL of the processed sample was eluted on an ACQUITY BEH C18 column (1.7 μm, 100 mm × 2.1 mm) (Waters, Milford, MA) by using 0.01% formic acid in water (mobile phase A) and acetonitrile (mobile phase B) as mobile phases. The flow rate was 0.45 mL/min with the following gradient: 0.0–0.5 min (95% A), 0.5–1.0 min (95%–64% A), 1.0–2.0 min (64%–74% A), 2.0–4.0 min (74%–70% A), 4.0–6.0 min (70% A), 6.0–7.0 min (70%–62% A), 7.0–9.0 min (62%–55% A), 9.0–12.5 min (55%–30% A), 12.5–13.0 min (30%–0% A), 13.0–14.0 min (0% A), 14.0–14.1 min (0%–95% A), and 14.1–15.0 min (95% A). The mass spectrometer was operated in the negative mode with capillary voltage of 3.0 kV, source temperature of 150°C, desolvation temperature of 550°C, cone gas flow of 150 L/h, and desolvation gas flow of 950 L/h. SIR and MRM transitions used to detect endogenous bile salts were described previously (Zhu et al., 2018). The calibration samples (4 nM–3,000 nM) and quality control samples (30 nM, 300 nM, and 3,000 nM) prepared in phosphate buffers were allocated into bioanalytical runs. The data of bile salts with plasma levels lower than the quantification limit in most samples were not reported.

### 2.5 Data processing and statistical analysis

The LC-MS raw data were processed by UNIFI (V1.8, Waters, Milford, MA, United States). Pharmacokinetic parameters were calculated using Phoenix^™^ WinNonlin^®^ software (V7.0, Pharsight) using a non-compartment model. Statistics of the exposure data was conducted after logarithmic transformation using GraphPad Prism software (V7.0, GraphPad Software, La Jolla, CA).

## 3 Results

### 3.1 Baseline is comparable between the fasting and fed studies

No difference in the population data was found for the 36 and 31 subjects who completed the fasting study and the fed study (Table 1). There was also no difference in the baseline of UDCA, GUDCA, and TUDCA between the two-treatment periods in both studies (data not shown), indicating the 28-day washout period was adequate to avoid the carryover effects of the first dosing on the second dosing visit. We then compared the baseline bile acid profiles acquired at the dosing visit of Ursofalk administration between the fasting study and the fed study. A total of 17 bile salts were determined in the −48 h∼0 h baseline plasma samples, including 10 downstream metabolites of CDCA (CDCA, GCDCA, TCDCA, UDCA, GUDCA, TUDCA, LCA, GLCA, GαHCA, and isoUDCA) and seven downstream metabolites of CA (CA, GCA, DCA, GDCA, TDCA, isoDCA, and 12-epiDCA). The metabolism pathways of them are illustrated in Figure 1. According to the molar percentage of the AUC data (Supplementary Figure S1), the baseline composition of plasma bile salts was consistent in the two studies, with the percentage decreasing in the order CDCA > DCA > CA > UDCA > LCA. A metabolite of UDCA, isoUDCA, accounted for 9.94% and 7.36% of the baseline AUC in the fasting and fed studies, respectively. As shown in the baseline variations within the 48 h baseline phase (Supplementary Figure S2), the high-fat diets given prior to the −48 h, −24 h, and 0 h sample collection time points induced bile excretion and caused a postprandial elevation of conjugated bile acids in the fed study. As a result, the average baseline of CDCA, GCDCA, TCDCA, GCA, and TDCA significantly increased, while the average baseline of isoDCA significantly decreased in the fed study. However, no significant difference was found for the baseline of UDCA, GUDCA, and TUDCA between the fasting study and the fed study, indicating a comparable baseline of bile salt metabolism in the two studies.

**TABLE 1:** Characteristics of subjects in the fasting and fed studies. All values represent the median including the range.

Study	Number, male/female	Age (years)	Weight (kg)	Height (cm)	BMI (kg/m^2^)
Fasting	36, 33/3	25.5 (18.0–51.0)	64.7 (49.2–77.8)	168.5 (148.5–185.5)	23.1 (19.3–25.6)
fed	31, 28/3	26.0 (18.0–53.0)	65.5 (51.4–83.5)	167.5 (152.5–180.5)	22.0 (20.1–25.9)

### 3.2 High-fat diets significantly delay and slightly increase UDCA absorption

**FIGURE 2:**
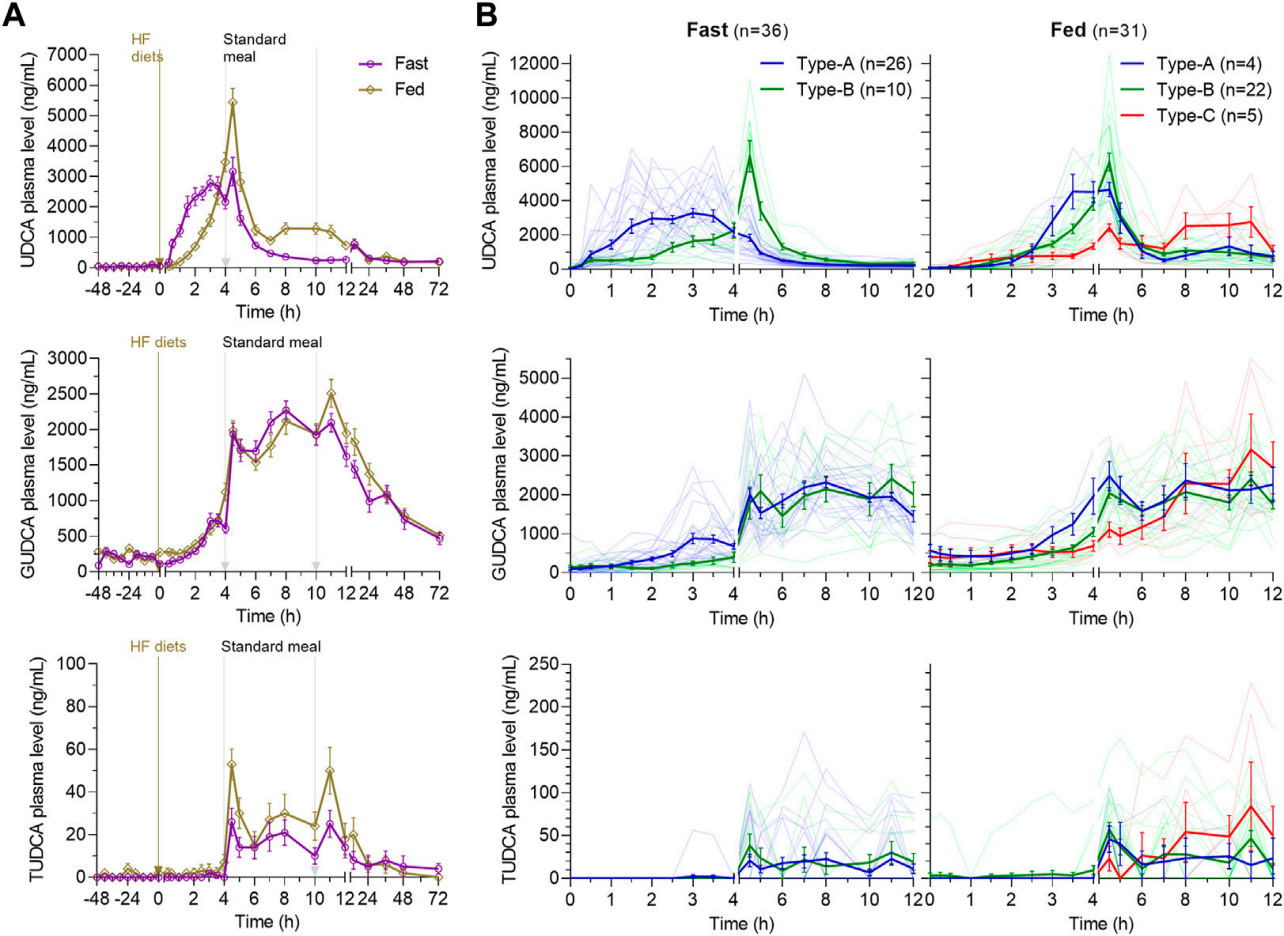
Average plasma concentration–time curves of UDCA, GUDCA, and TUDCA in the fasting study and the fed study **(A)** and the three absorption types of subjects categorized according to the time to reach the maximum concentration of UDCA **(B)**. The data are shown as the mean ± SEM. Abbreviations: UDCA, ursodeoxycholic acid; GUDCA, glycoursodeoxycholic acid; and TUDCA, tauroursodeoxycholic acid.

As shown in Figure 2A, the multiple peaks of UDCA in the average plasma concentration–time (C–T) curve indicated diverse absorption processes under the fast or fed condition. In the fasting study, UDCA was quickly absorbed with the first peak at 0 h∼4 h under the fasting state and another peak at 4.5 h following the standard lunch given at 4 h. In the fed study, the absorption of UDCA was delayed by the high-fat diet with the peak reaching 4.5 h following the standard lunch. The absorption processes were inspected in detail by looking into the individual C–T curve of UDCA within 0 h–12 h. As shown in Figure 2B, the subjects in both studies were categorized into three absorption types. Type-A has T_max_ within 0 h∼4 h, indicating that the absorption occurs primarily before the standard lunch. Type-B has the delayed T_max_ at 4.5 h, indicating a facilitated absorption by the standard lunch. Type-C has severely delayed T_max_ more than 6 h. In the fasting study, 26 of 36 subjects (72%) demonstrated Type-A absorption and 10 subjects exhibited Type-B absorption. In the fed study, 22 of 31 subjects (71%) demonstrated Type-B absorption and five subjects exhibited heavily delayed Type-C absorption. The data indicated a delayed absorption by the HF diets and clarified that the second peak in the average C-T curves was caused by individual difference in the absorption types.

Consistent with the fact that bile acids mainly conjugate with glycine in adults, the absorbed UDCA is conjugated primarily into GUDCA and in traces into TUDCA (Figure 2A). The data of TUDCA were not separately analyzed but incorporated into the total UDCA data (total UDCA is defined as the sum of UDCA, GUDCA, and TUDCA in standard with the UDCA molecular weight). GUDCA and TUDCA had atypical pharmacokinetic C–T curves driven by enterohepatic circulation, as shown by multiple peaks between the standard meals. In both studies, the pharmacokinetics of GUDCA within 0 h∼4 h was well consistent with the absorption types of UDCA (Figure 2B). In the fasting and fed studies, the UDCA baseline accounted for 0.9% (0.0%–7.3%) and 1.0% (0.0%–7.1%) of the C_max_ of UDCA with no statistical difference (*p* = 0.83), respectively. The GUDCA baseline accounted for 5.7% (0.0%–20.1%) and 7.2% (0.0%–21.1%) of the C_max_ of GUDCA with no difference (*p* = 0.23). Therefore, the baseline corrected PK parameters of UDCA, GUDCA, and total UDCA were then compared between the two studies to present the food effects on the pharmacokinetics of UDCA.

Table 2 lists the baseline-corrected pharmacokinetic parameters of UDCA, GUDCA, and total UDCA, in which the AUC_0–4h_, AUC_4–12h_ and AUC_12–72h_ were separately calculated according to the absorption types illustrated in Figure 2B. The MRT rather than terminal half-life was provided due to the atypical elimination phase of UDCA, GUDCA, and total UDCA. For UDCA, plasma concentrations peaked with the median T_max_ of 3.3 h in the fasting study, which was significantly faster than the median T_max_ of 4.5 h in the fed study (*p* < 0.0001). As a result, compared to the fasting study, the AUC_0–4h_ of UDCA in the fed study significantly decreased, but the AUC_4–12h_ of UDCA significantly increased, respectively, while the AUC_12–72h_ of UDCA showed no difference in both studies. However, the C_max_ of UDCA showed no difference with geometric mean data of 5.215 μg/mL and 5.555 μg/mL in the fasting and fed study, respectively. The delayed absorption of UDCA by the high-fat diet resulted in a 21% increase (*p* = 0.016) in the geometric mean data of AUC_0–72h_ of UDCA in the fed study (30.8 μg·h/mL) compared with the fasting study (25.4 μg·h/mL). On the other hand, the baseline-corrected pharmacokinetic parameters of GUDCA remained unchanged in both studies except for the T_max_, which was delayed to the median data of 10.0 h in the fed study from 8.0 h in the fasting study. Combining the data of UDCA, GUDCA, and TUDCA, total UDCA showed significant differences in the T_max_ (*p* < 0.0001) and C_max_ (*p* = 0.0138). However, the AUC_0–72h_ of total UDCA demonstrated a non-significant increase from 73.0 μg·h/mL in the fasting study to 83.4 μg·h/mL in the fed study, which was clearly explained by the reverse differences in AUC_0–4h_ and AUC_4–12h_ between the two studies.

**TABLE 2:** Summary of the baseline-corrected pharmacokinetic parameters of UDCA, GUDCA, and total UDCA. The data are shown as the geometric mean/CV% (range).

Parameter	Fasting (*N* = 36)		Fed (*N* = 31)		Statistics*
UDCA					
Baseline (ng/mL)	51/180.1	(0.0–362.9)	47/128.7	(0.0–198.5)	ns
Baseline/Cmax (%)	0.9/174	(0.0–7.3)	1.0/153	(0.0–7.1)	
T_max_ (h)	3.3	(0.5–14.0)	4.5	(3.0–11.0)	<0.0001
C_max_ (ng/mL)	5,215/35.3	(2,426–10,829)	5,555/40.4	(2,359–12,613)	ns
AUC_0–4h_ (ng·h/mL)	6,581/46.6	(1,007–13,796)	3,371/58.4	(650–8,442)	<0.0001
AUC_4–12h_ (ng·h/mL)	4,593/72.4	(1,312–13,784)	12,302/32.7	(6,876–23,337)	<0.0001
AUC_12–72h_ (ng·h/mL)	11,552/74.2	(3,118–40,632)	13,396/59.8	(3,118–41,597)	ns
AUC_0–72h_ (ng·h/mL)	25,385/36.3	(14,053–53,116)	30,753/32.3	(18,896–66,911)	0.016
MRT_0–72h_ (h)	18.0	(9.2–30.1)	18.2	(8.9–26.4)	ns
GUDCA					
Baseline (ng/mL)	189/115.0	(0.0–835.8)	232/82.1	(0–738)	ns
Baseline/Cmax (%)	5.7/90	(0.0–20.1)	7.2/73	(0.0–21.1)	
T_max_ (h)	8.0	(3.5–36.0)	10.0	(4.5–24.0)	<0.0001
C_max_ (ng/mL)	2,619/30.6	(809–4,392)	2,825/33.0	(1818–5,786)	ns
AUC_0–4h_ (ng·h/mL)	664/138.4	(0–3,468)	699/92.3	(30–2,236)	ns
AUC_4–12h_ (ng·h/mL)	13,329/26.8	(3,718–20,799)	12,915/33.7	(5,812–22,917)	ns
AUC_12–72h_ (ng·h/mL)	37,335/71.2	(14,921–169,433)	43,946/44.7	(18,703–102,020)	ns
AUC_0–72h_ (ng·h/mL)	52,342/54.5	(18,900–190,652)	58,490/38.4	(29,938–125,141)	ns
MRT_0–72h_ (h)	26.1	(15.2–36.1)	26.2	(17.3–34.0)	ns
Total UDCA [=(UDCA/392.6 + GUDCA/449.6 + TUDCA/499.7)*392.6]					
T_max_ (h)	3.5	(0.5–14.0)	4.5	(3.5–11.0)	<0.0001
C_max_ (ng/mL)	6,022/38.2	(2,706–13,343)	7,405/31.6	(4,235–13,862)	0.0138
AUC_0–4h_ (ng·h/mL)	7,232/50.3	(1,007–14,232)	4,070/59.6	(677–9,731)	<0.0001
AUC_4–12h_ (ng·h/mL)	17,052/28.7	(8,901–31,206)	24,220/26.1	(16,617–42,614)	<0.0001
AUC_12–72h_ (ng·h/mL)	46,342/56.6	(23,491–163,702)	53,511/38.6	(22,295–111,247)	ns
AUC_0–72h_ (ng·h/mL)	73,038/39.0	(46,644–195,961)	83,437/29.9	(47,083–155,647)	ns
MRT_0–72h_ (h)	22.5	(13.1–33.7)	23.5	(17.3–30.5)	ns

*Unpaired *t* tests were performed for the exposure data after logarithmic transformation. A Wilcoxon signed-rank test was performed for T_max_ and MRT data.

### 3.3 High-fat diets elevate circulating bile salts and attenuate UDCA replacement

**FIGURE 3:**
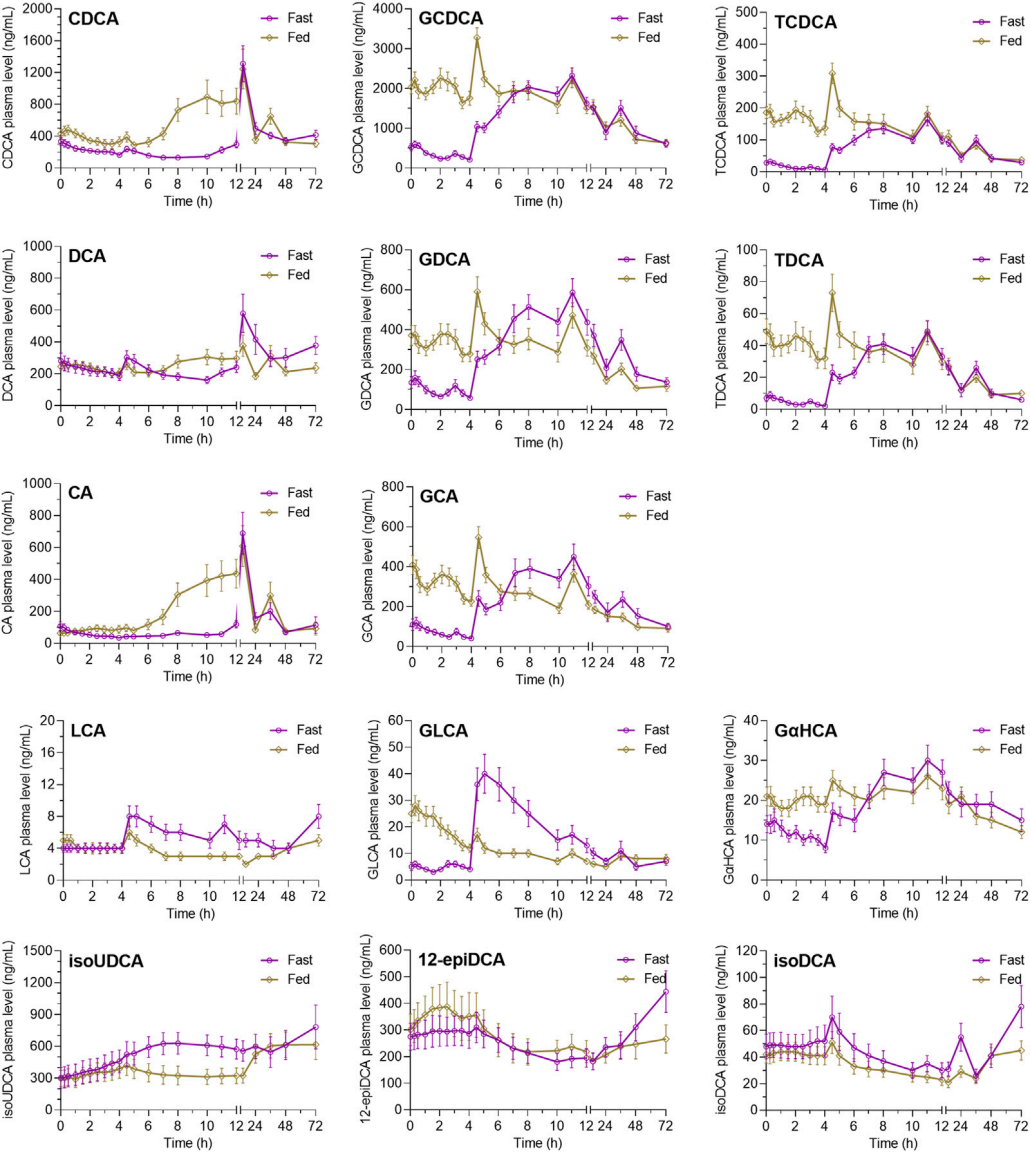
Average plasma concentration–time curves of endogenous bile salts after oral UDCA administration in the fasting study and the fed study. The data are shown as the mean ± SEM. Abbreviations: CA, cholic acid; CDCA, chenodeoxycholic acid; DCA, deoxycholic acid; GCA, glycocholic acid; GCDCA, glycochenodeoxycholic acid; GDCA, glycodeoxycholic acid; GLCA, glycolithocholic acid; GUDCA, glycoursodeoxycholic acid; isoUDCA, isoursodeoxycholic acid; LCA, lithocholic acid; TCDCA, taurochenodeoxycholic acid; TDCA, taurodeoxycholic acid; UDCA, ursodeoxycholic acid; 12-epiDCA, 3α, 12β-dihydroxy-5β-cholan-24-oic acid; isoDCA, isodeoxycholic acid; and GαHCA, 7α-glycohyocholic acid.

As shown in Figure 3, the post-dose variations of endogenous bile salts in circulation also showed diet-associated patterns that are different in the fasting study and the fed study. For the conjugated bile acids, including GCDCA, GDCA, GCA, GLCA, TCDCA, TDCA, and GαHCA, the fed study demonstrated significantly increased plasma levels at 0 h–4 h post-dose compared to the fasting study, which is consistent with the diet-induced bile excretion. Subsequently, the difference gradually decreased after the standard lunch and disappeared after the standard dinner on the dosing day. For the unconjugated bile acids, such as CDCA, DCA, and CA, the plasma levels of them at 0 h–4 h post-dose were not elevated by the high-fat diets as significantly as the conjugated bile acids. However, after the standard lunch given at 4 h post-dose, the plasma levels of CDCA, DCA, and CA gradually increased in the fed study compared to the fasting study, while the increase disappeared after the standard dinner on the dosing day. IsoUDCA, the microbial 3-epimerized metabolite of UDCA, demonstrated a reversed post-dose variation pattern compared to CDCA, DCA, and CA. The plasma levels of LCA, another microbial metabolite of UDCA, were too low to be interpreted despite its variation pattern seeming to be similar as that of isoUDCA.

**FIGURE 4:**
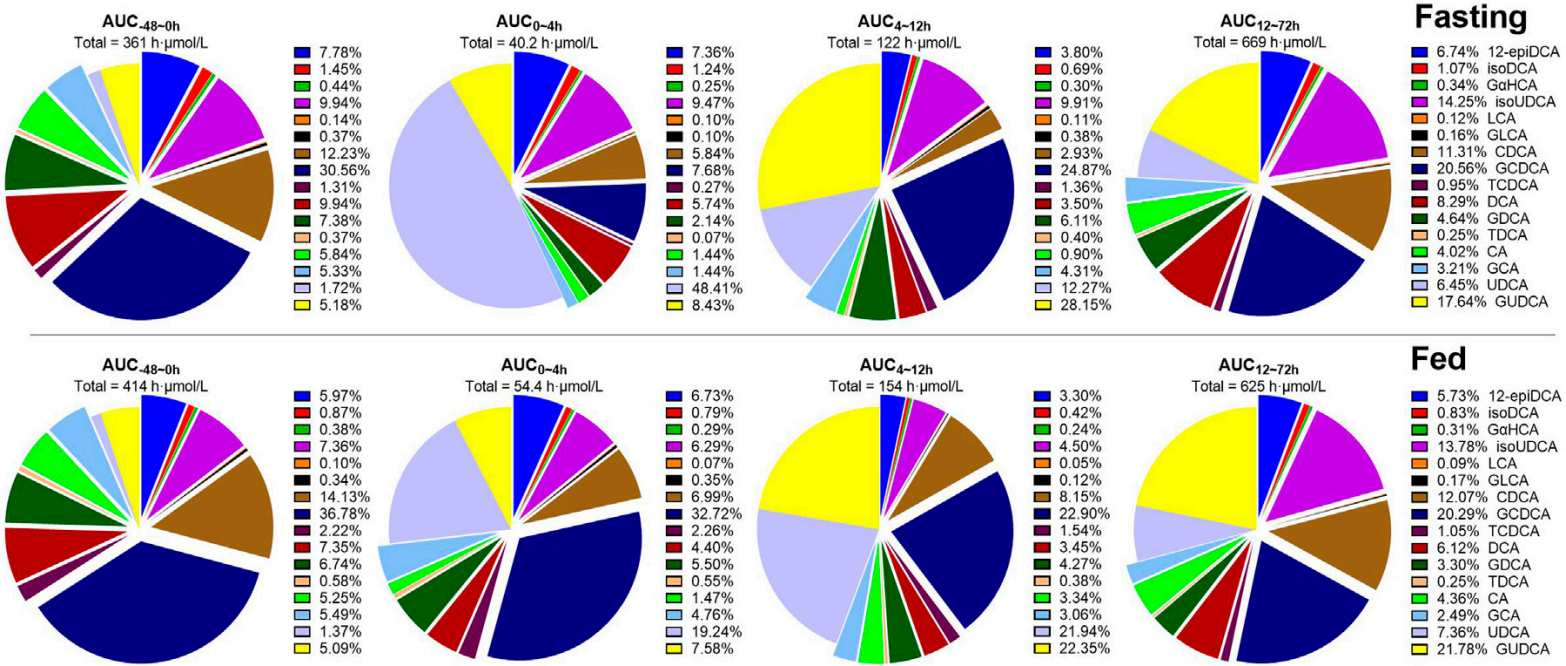
Molar percentages of circulating bile salts calculated according to AUC_-48-0h_, AUC_0–4h_, AUC_4–12h_, and AUC_12–72h_ of 16 bile salts. Abbreviations: CA, cholic acid; CDCA, chenodeoxycholic acid; DCA, deoxycholic acid; GCA, glycocholic acid; GCDCA, glycochenodeoxycholic acid; GDCA, glycodeoxycholic acid; GLCA, glycolithocholic acid; GUDCA, glycoursodeoxycholic acid; isoUDCA, isoursodeoxycholic acid; LCA, lithocholic acid; TCDCA, taurochenodeoxycholic acid; TDCA, taurodeoxycholic acid; UDCA, ursodeoxycholic acid; 12-epiDCA, 3α, 12β-dihydroxy-5β-cholan-24-oic acid; isoDCA, isodeoxycholic acid; and GαHCA, 7α-glycohyocholic acid.

According to the diet-associated variation patterns, the molar percentage of plasma bile salts is summarized in Figure 4 according to the data of AUC_-48-0h_, AUC_0–4h_, AUC_4–12h_, and AUC_12–72h_. In both studies, UDCA and GUDCA replaced the hydrophobic bile salts in circulation. The most significant replacement was observed within 0 h–4 h post-dose in the fasting study, which was much higher than the corresponding data in the fed study. The faster absorption of UDCA under the fasting state and the higher circulating bile salts under the fed state simultaneously contributed to the significant attenuation of UDCA replacement by HF diets. After the standard meals given after UDCA administration, both studies demonstrated similar extents of UDCA replacements. Meanwhile, the increased percentages of isoUDCA, the major microbial metabolite of UDCA, were also observed in both studies, mainly in the data of AUC_12–72h_, which is well consistent with the fact that the metabolic reactions occur mainly in the lower gut where bacteria colonize.

## 4 Discussion

UDCA is an endogenous bile salt, which is quickly conjugated mainly with glycine as GUDCA in hepatocytes after absorption in adults and is taken up into the gallbladder bile and undergoes continuous enterohepatic circulation between meals. Although the therapeutic efficacy of UDCA medication is well correlated with the replacement percentage of UDCA metabolites in biliary bile salts, it is hard to collect the gallbladder bile samples for bile salts composition analysis in clinical practice. Despite the plasma levels being merely reflective of the overflow of bile salt pool into blood circulation, the pharmacokinetic analysis serves as a practical indicator for UDCA utilization. As shown in Figure 1, it has to be mentioned that GUDCA is readily uptaken by bile salts transporters, such as sodium taurocholate co-transporting polypeptide, canalicular bile salt export pump, apical sodium-dependent bile salt transporter, and organic solute transporter (Halilbasic et al., 2013). This work, therefore, found no difference between the fasting and fed studies for the exposure data of GUDCA except for T_max_. In contrast, the absorption phase of UDCA is sensitive to the dietary effects because it is less susceptive to bile salt transporters than GUDCA.

UDCA is a lipophilic drug with alkaline pH-dependent solubility. As shown in Supplementary Table S2, we determined the thermodynamic solubilities of UDCA by shaking flask methods at 37°C in 0.1M HCl, pH 4.0 acetate buffer, and pH 6.5, 6.8, 7.2, 7.5, and 8.0 phosphate buffers. The data were 1.9, 1.8, 120, 250, 530, 950, and 1,600 μg/mL, respectively, indicating that the absorption of UDCA is dissolution limited in the gastrointestinal tract. The food effects on UDCA utilization are anticipated to be associated on one hand with the gastric emptying time and on the other hand with the solubilization of UDCA by emulsification with both diet lipids and endogenous bile micelles. Corresponding to the expectation, we observed that the HF diets significantly delayed the absorption of UDCA most probably by retaining the dose in the stomach, in which UDCA hardly dissolves in the acidic gastric fluid. After the standard lunch given at 4 h after dose, once the retained dose was transferred from the stomach to the small intestine, in which the solubility of UDCA gradually increases, a marked elevation of plasma UDCA levels was observed only in the fed study but not in the fasting study. The exposure of UDCA was slightly enhanced in the fed study, which was probably associated with the solubilization of UDCA by the HF diet. This phenomenon reinforced the proposal that the absorption of UDCA is primarily limited by the empty gastric condition and may be additionally enhanced by solubilization effects of diet lipids.

Accompanying the altered UDCA absorption, the disposition of endogenous bile salts was also markedly perturbed by the food effects. The HF diets heavily induce bile secretion, resulting in sharp increases of circulating GCDCA, GDCA, GCA, GLCA, TCDCA, and TDCA compared to the fasting study. It was worth mentioning that the plasma levels of CDCA, DCA, and CA remained stable in the fasting study within the two standard meals given on the dosing day. In contrast, the circulating levels of them gradually increased at the same time window in the fed study, which may be explained by the fact that large amounts of GCDCA, GDCA, and GCA escape intestinal uptake and enter the lower gut, where they will be hydrolyzed into CDCA, DCA, and CA and recovered into circulation. Another noteworthy observation was about the microbial metabolism of UDCA into isoUDCA. The baseline plasma level of isoUDCA was unexpectedly higher than that of UDCA, indicating that UDCA is inclined to be 3-epimerized into isoUDCA. Consistent with the fact that the epimerization reaction occurs in the lower gut, the plasma levels of isoUDCA were resistant to bile secretion induced by diet interventions. Accordingly, the plasma levels of isoUDCA had no difference at 0 h–4 h post-dose in both studies, while the gradual increases did not appear until the undissolved UDCA reached the lower gut, finally resulting in the increased percentages of isoUDCA in the circulating bile salt pool in both studies.

The hydrophobic bile acids reported in the literature so far mainly include the following: LCA, DCA, and CDCA and conjugates GDCA, TDCA, GCDCA, and TCDCA (Gu et al., 1992; Auwerx et al., 2008). In addition, some studies also treat CA as hydrophobic bile acids although CA has three hydroxyl groups and its solubility is even higher than that of GUDCA in some literature; however, its hydroxyl groups are all in α-configuration. Six of the 17 endogenous bile acids finally quantified had significant differences in level of baseline between the two studies, four of them (CDCA, GCDCA, TCDCA, and TDCA) were hydrophobic bile acids, and of the other two bile acids with differences, GCA had a solubility similar to that of GUDCA (Zhu et al., 2018), and isoDCA had increased hydrophilicity with its 3-hydroxyl undergoing β-isomerization (Gu et al., 1992), which can be considered as a hydrophilic bile acid. Overall, it was seen that the major hydrophobic bile acids in circulating bile acids were more susceptible to high-fat meals in both fasting and fed studies, with significantly higher concentrations in the circulation, while baseline concentrations of hydrophilic bile acids did not differ between the two studies, with only isoDCA exhibiting higher baseline concentrations in the fasting study. Biliary excretion induced by high-fat meals resulted in elevated circulating conjugated bile acids mainly acting on hydrophobic bile acids.

## 5 Conclusion

In conclusion, this work reported how the HF diets affect the absorption and disposition of UDCA and how the circulated levels of endogenous bile salts are simultaneously perturbed in healthy subjects. It was disclosed that the HF diets significantly delay UDCA absorption due to the extension of gastric empty time, which is the determinant for UDCA dissolution. Although the HF diets may slightly facilitate the UDCA absorption probably *via* solubilization effects, the beneficial effect may be limited because the HF diets also heavily induced bile secretion, leading to sharp increases of circulating hydrophobic bile salts, some of which are recently revealed to be correlated to cholestatic itch (Yu et al., 2019). The present findings should be carefully translated into clinical practices because this study conducted in healthy population is not a self-reference study specifically designed to investigate the food effects in patients. However, due to the complexity of bile salt metabolism, the preliminary results raise the necessity of randomized controlled trials to clarify the food effects on the benefits and risks of UDCA treatments for the sake of cholestatic patients.

## Data Availability

The original contributions presented in the study are included in the article/Supplementary Material; further inquiries can be directed to the corresponding authors.
