# Supplementary material for: High-fat diets enhance and delay ursodeoxycholic acid absorption but elevate circulating hydrophobic bile salts
Source: Front Pharmacol. 2023 Apr 17;14:1168144. doi: 10.3389/fphar.2023.1168144 (PMC10149867; doi:10.3389/fphar.2023.1168144)
Supplement: Supplementary file 1 [file DataSheet1.pdf]

Supplementary Material

High Fat Diets Enhance and Delay Ursodeoxycholic Acid Absorption but Elevate Circulating Hydrophobic Bile Salts

Liang Huang†**, Wei Wei**†

*** Correspondence:** Bikui Zhang, bikui_zh@126.com, Ke Lan, lanwoco@scu.edu.cn,

1 Supplementary Figures

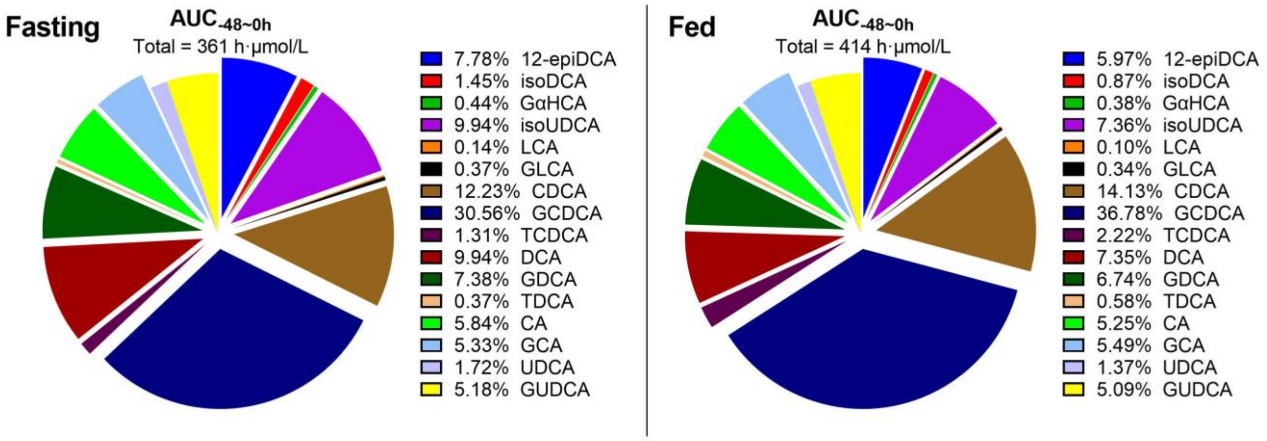

Supplementary Figure 1. The molar percentage of baseline AUC data of 16 circulating bile salts is consistent in the fasting study and the fed study.

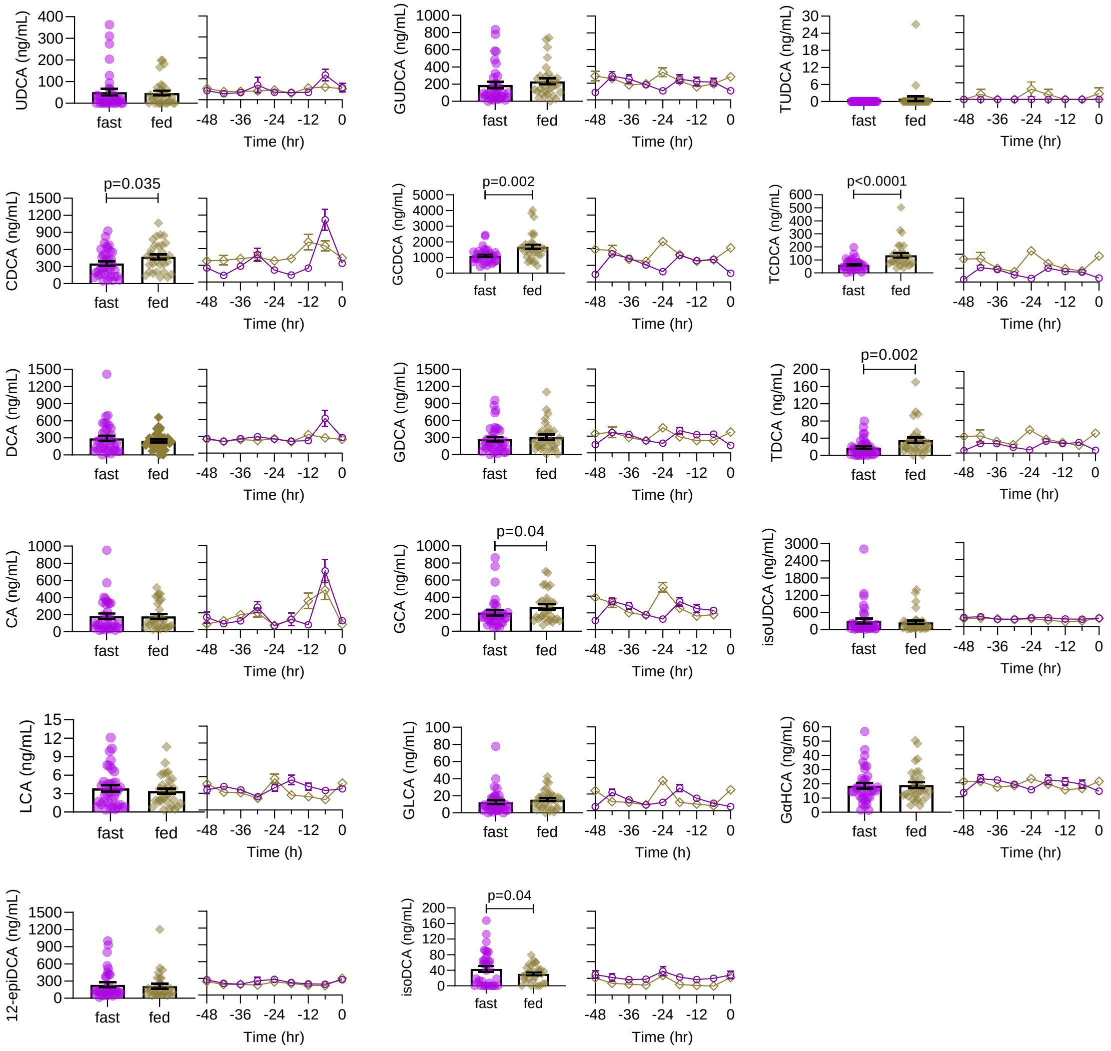

Supplementary Figure 2. The average baseline levels of 17 circulating bile salts and their variations during the -48~0h baseline period in the fasting study and the fed study. Data was shown as mean ± SEM. Unpaired *t* tests were performed after logarithmic transformation

2 Supplementary tables

Supplementary table1 The NO. abbreviation, name, quantitative LC-MS profiling method of 26 bile acids.

NO.	Abbreviation	Name	Category	quantitative LC-MS profiling method		
				MRM transient	Retention time (min)	Internal standard
01	LCA	Lithocholic acid	Unconjugated	375>375	12.8	LCA-D4
02	isoUDCA	Isoursodeoxycholic acid	Unconjugated	391>391	8.4	UDCA-D4
03	UDCA	Ursodeoxycholic acid	Unconjugated	391>391	8.8	UDCA-D4
04	HDCA	Hyodeoxycholic acid	Unconjugated	391>391	9.0	UDCA-D4
05	12-epiDCA	12-epideoxycholic acid	Unconjugated	391>391	9.5	DCA-D4
06	isoDCA	Isodeoxycholic acid	Unconjugated	391>391	9.7	DCA-D4
07	CDCA	Chenodeoxycholic acid	Unconjugated	391>391	10.8	CDCA-D4
08	DCA	Deoxycholic acid	Unconjugated	391>391	11.0	DCA-D4
09	UCA	Ursocholic acid	Unconjugated	407>407	4.4	CA-D4
10	isoCA	Isocholic acid	Unconjugated	407>289	6.2	CA-D4
11	αHCA	Hyocholic acid	Unconjugated	407>407	8.1	CA-D4
12	CA	Cholic acid	Unconjugated	407>407	8.7	CA-D4
13	GLCA	Glycolithocholic acid	Glycine conjugate	432>74	11.2	GLCA-D4
14	GUDCA	Glycoursodeoxycholic acid	Glycine conjugate	448>74	4.2	GUDCA-D4
15	GHDCA	Glycohyodeoxycholic acid	Glycine conjugate	448>74	4.4	GUDCA-D4
16	GCDCA	Glycochenodeoxycholic acid	Glycine conjugate	448>74	7.7	GCDCA-D4
17	GDCA	Glycodeoxycholic acid	Glycine conjugate	448>74	8.1	GDCA-D4
18	GαHCA	Glycohyocholic acid	Glycine conjugate	464>74	4.5	GCA-D4
19	GCA	Glycocholic acid	Glycine conjugate	464>74	3.0	GCA-D4
20	TLCA	Taurolithocholic acid	Taurine conjugate	482>80	10.2	TLCA-D4
21	TUDCA	Tauroursodeoxycholic acid	Taurine conjugate	498>80	4.5	TUDCA-D4
22	THDCA	Taurohyodeoxycholic acid	Taurine conjugate	498>80	4.7	TUDCA-D4
23	TCDCA	Taurochenodeoxycholic acid	Taurine conjugate	498>80	8.2	TCDCA-D4
24	TDCA	Taurodeoxycholic acid	Taurine conjugate	498>80	8.9	TDCA-D4
25	TαHCA	Taurohyocholic acid	Taurine conjugate	514>80	3.3	TCA-D4
26	TCA	Taurocholic acid	Taurine conjugate	514>80	5.3	TCA-D4

Supplementary table 2. the thermodynamic solubilities of UDCA. Data determined by shaking flask methods at 37 °C.

Solution	pH	Solubility (ug/ml)
0.1M HCl	1.0	1.9
Acetic acid buffer	4.0	1.8
	6.5	120
	6.8	250
Phosphate buffer	7.2	530
	7.5	950
	8.0	1600
